# Structure evolution of nanodiamond aggregates: a SANS and USANS study

**DOI:** 10.1107/S1600576722002084

**Published:** 2022-03-25

**Authors:** Imrana I. Kabir, John C. Osborn, Weijian Lu, Jitendra P. Mata, Christine Rehm, Guan H. Yeoh, Tunay Ersez

**Affiliations:** a School of Mechanical and Manufacturing Engineering, University of New South Wales, Sydney, NSW 2052, Australia; b Australian Nuclear Science and Technology Organisation, Locked Bag 2001, Kirrawee DC, NSW 2232, Australia

**Keywords:** nanodiamonds, cold neutron sources, detonation NDs, small-angle neutron scattering, laser synthesis technique

## Abstract

Ultra-small-angle neutron scattering (USANS) and small-angle neutron scattering (SANS) measurements, covering length scales from micrometres to nanometres, were made to investigate the structure of nanodiamonds (NDs) and their suspensions. These nanodiamonds were produced by two different techniques, namely by the detonation method and by the laser ablation of a carbon–hydro­carbon mixture. The (U)SANS results indicated the presence of structures four orders of magnitude larger than the dimensions of a single ND particle, consisting of aggregations of ND particles. This aggregation of the ND particles was studied by employing the contrast variation technique. Two different solvents, namely H_2_O and dimethyl sulfoxide (and their deuterated counterparts), were used to understand the role of hydrogen in the shape and size of the aggregates. The analysis of experimental data from SANS measurements also reveals the ND particles to have an ellipsoidal structure. Using a defined shape model and the SANS contrast variation technique, it was possible to characterize the non-diamond outer shell of the particles and determine the outer layer thickness. This clarification of the structure of the NDs will allow better preparation of suspensions/samples for various applications. Understanding the structure of NDs at multiple length scales also provides crucial knowledge of particle–particle interaction and its effect on the aggregation structures.

## Abstract 2

Nanodiamonds (NDs) produced by two different techniques have been studied by ultra-small- (USANS) and small-angle neutron scattering (SANS): (i) produced by the detonation of explosives in an oxygen-deficient atmosphere (TNT + hexogen), which results in the production of detonation nanodiamonds, and (ii) produced by laser ablation of a carbon–hydro­carbon mixture. The size distribution of the particles and the composition of the outer layers are expected to vary depending on the source of the NDs.

## 1. Introduction

Nanodiamond (ND) materials consist of particles with a diamond core surrounded by an outer layer of graphitic carbon and organic material, with a typical total radius of 25–35 Å (Fang *et al.*, 2009 ▸; Panich *et al.*, 2010 ▸; Baidakova *et al.*, 2013 ▸). ND powders are good reflectors of very cold neutrons (VCNs) (Lychagin *et al.*, 2009 ▸; Nesvizhevsky, Dubois *et al.*, 2018 ▸). For this reason, a number of applications of NDs in cold neutron technology have been suggested (Nesvizhevsky *et al.*, 2010 ▸): (i) a blanket of ND powder surrounding a cold neutron source (CNS) or a cold neutron trap may reduce VCN losses through the wall by reflecting VCNs back into the source or trap (Ersez *et al.*, 2018 ▸), and (ii) ND powder may be used as a reflective surface in a novel type of neutron guide (Nesvizhevsky, Dubois *et al.*, 2018 ▸). NDs have also been used in a variety of other fields, such as in fine polishing, lubricating, coatings and polymers. Initially, NDs were used in the defence industry, but currently they have applications in biomedicine, thermal management in electronics, photovoltaics and energy storage (Zousman & Levinson, 2012 ▸).

Detonation ND (DND) powders are produced by the detonation of explosives in an oxygen-deficient atmosphere (TNT + hexogen). They tend to form clusters and consolidate into larger multiscale aggregates (agglomerations can be >1000 Å in size), similar to those exhibited by other powdery nanomaterials. ND aggregates of <1000 Å have been found to form (during explosion conditions) with C—C bonds between the primary nanoparticles. Other types of ND aggregates form through the creation of bonds between oxygen-containing surface groups and van der Waals forces during the ND purification stages (Popov, 2021 ▸). Several groups of researchers have investigated various mechanical and chemical techniques to reduce the agglomeration of DNDs and substitute functional groups on their surface (Krueger & Boedeker, 2008 ▸; Aleksenskiy *et al.*, 2011 ▸; Shvidchenko *et al.*, 2017 ▸).

**Figure 1:**
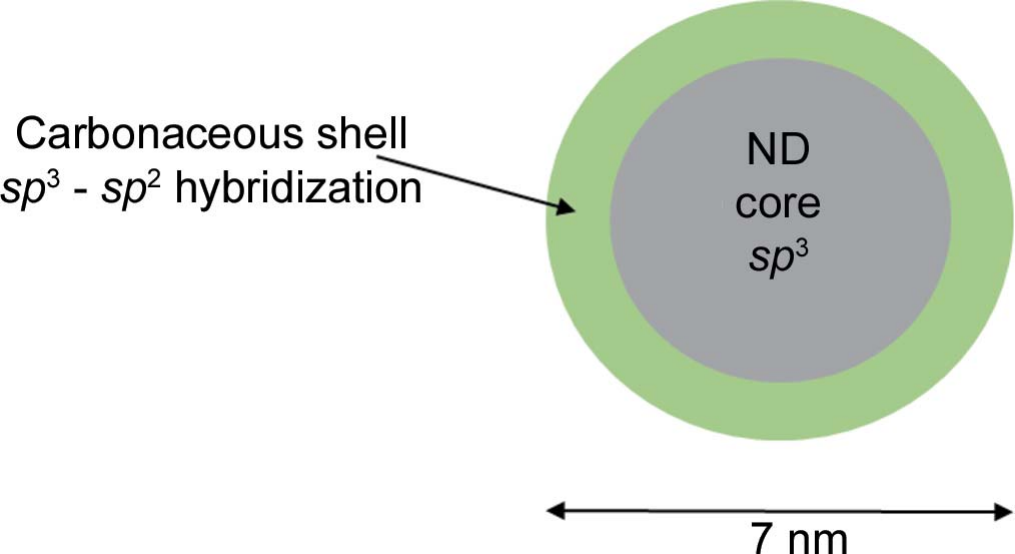
A schematic model of the structure of an ND.

NDs may be produced by several methods (Baidakova *et al.*, 2013 ▸). Proposed applications of NDs for cold neutron containment or reflection have so far focused on DNDs, and a number of neutron scattering studies have been conducted on DNDs (Aleksenskii *et al.*, 2021 ▸). DNDs have provided record reflectivity compared with other known media, for example the reflection probability far exceeds the corresponding values for available supermirrors [see Fig. 9 in the work of Lychagin *et al.* (2009 ▸)]. The diamond core has been assumed to have *sp* ^3^ hybridization with a polyhedron shape, and there is a non-crystalline carbonaceous shell having *sp* ^3^–*sp* ^2^ hybridization of C atoms surrounding the core (Barnard & Sternberg, 2007 ▸) (see Fig. 1 ▸).

The most efficient methods applied in structural research on ND suspensions have been the techniques of neutron and X-ray scattering, especially ultra-small-angle and small-angle neutron scattering (USANS and SANS, respectively) and small-angle X-ray scattering (SAXS), and without knowledge of the structure of NDs it would be difficult to tailor materials for certain applications. These techniques, especially with the option of using contrast variation based on isotopic hydrogen/deuterium substitution in solvents, have the ability to determine various phases of aggregates (Tomchuk, Avdeev *et al.*, 2019 ▸). The contrast variation is generated by preparing suspensions of NDs in mixtures of light (hydrogenous) and heavy (deuterated) solvents. The changes in the scattering curves with different contents of the deuterated component in the liquid carrier allow analysis of the NDs. It is widely reported that NDs have a shell that is less dense than the core, which is responsible for the stability of ND suspensions. It is essential to understand the core–shell structure to produce stable suspensions of NDs in water. By using H_2_O and D_2_O one can contrast match the shell (see Fig. 5 below). The scattering has been observed to decay monotonically with increasing deuterium content and evidence has been presented for the homogeneity of the aggregates under study (Ersez *et al.*, 2018 ▸; Tomchuk *et al.*, 2011 ▸; Avdeev *et al.*, 2009 ▸). Tomchuk *et al.* (2011 ▸) have also suggested that there is a graphene shell around the diamond core. The decrease in the subparticle radius of gyration (with decreasing contrast) from 4.6 (8) to 2.8 (5) nm is consistent with this conclusion, because the shell is partially shaded from the scattering viewpoint as the share of heavy water in the buffer increases.

Industrial DND powders tend to contain strongly connected, mostly dense, agglomerates with sizes of 400–2000 Å (Aleksenskii *et al.*, 2021 ▸). Therefore, U/SANS and SAXS investigations are expected to yield information on the size distribution of the diamond core and outer organic layer as well as the size of the particle aggregates (Avdeev *et al.*, 2009 ▸; Tomchuk *et al.*, 2014 ▸; Tomchuk, Volkov *et al.*, 2019 ▸). This information is required to model the neutron albedo of the material, which is important for proposed CNS applications and to improve the efficiency of neutron delivery to experimental installations, and also, for example, in targeted drug delivery.

Currently available NDs contain hydrogen in large quantities (Bosak *et al.*, 2020 ▸) in the outer shells of the particles, in the form of C—OH, C—H, CH_2_ and COOH (Mochalin *et al.*, 2012 ▸; Krueger, 2017 ▸). However, hydrogen in NDs is an important cause of neutron losses, reducing the efficiency of the reflection. Recently, a gas (F_2_) fluorination chemical treatment applied to DNDs reduced the quantity of hydrogen by a factor of ∼30, but the quantity of sample obtained was less than 50 mg (Nesvizhevsky, Koester *et al.*, 2018*a* ▸,*b* ▸; Herraiz *et al.*, 2020 ▸). Deuterated NDs have been found to be unstable relative to the substitution of D by H (Bosak *et al.*, 2020 ▸; Batsanov *et al.*, 2018 ▸).

NDs have also been produced by the laser synthesis technique, and these are termed LNDs. The technique was developed by Ray Techniques Ltd (Jerusalem, Israel) and is based on high-intensity laser radiation treatment of a specially prepared target containing non-diamond carbon soot and hydro­carbons, placed in a liquid medium (Zousman & Levinson, 2014 ▸, 2012 ▸). This results in carbon atoms forming a cubic diamond crystalline structure, determined using X-ray diffraction and by studying the crystal sizes (average 40–50 Å) using transmission electron microscopy. LNDs can have a higher level of purity and homogeneity than DNDs (Zousman & Levinson, 2014 ▸, 2012 ▸), and therefore have significant advantages for most ND applications.

In this work, NDs produced by these two different techniques have been studied, namely the detonation of explosives in an oxygen-deficient atmosphere (TNT + hexogen), which results in the production of detonation nanodiamonds (DNDs), and laser ablation of a carbon–hydro­carbon mixture, which produces LNDs. The size distribution of the particles and the composition of the outer layers are expected to vary depending on the source of the NDs, and as noted above LNDs can have smaller amounts of impurities. Our aim is to produce a standard structural model for NDs which could then be used to study various ND samples. A comparison of DNDs and LNDs is therefore of interest.

In order to understand these structures at multiple length scales (up to four orders of magnitude), we have combined USANS with SANS. Due to the extended scattering vector *Q* value [*Q* = (4π/λ)sin(θ/2), where θ is the scattering angle and λ is the wavelength of the incident neutrons] achieved by combining USANS and SANS, we are now able to correlate the effect of the nanostructure of NDs on their aggregation behaviour. The SANS component of our experiments is similar to work carried out by Avdeev and others (Zousman & Levinson, 2012 ▸; Tomchuk, Avdeev *et al.*, 2019 ▸; Tomchuk *et al.*, 2011 ▸; Avdeev *et al.*, 2009 ▸) on DNDs. Our earlier work on DNDs and LNDs (Ersez *et al.*, 2018 ▸) suggested the presence of structures with a much larger length scale than the dimensions of a single ND particle, and these have been shown to consist of aggregations of ND particles (Avdeev *et al.*, 2009 ▸). Such aggregation of these particles may affect the proposed applications, and also the potential industrial and medical applications of ND suspensions.

For contrast variation measurements we focused on NDs in H_2_O, D_2_O, and hydrogenated and deuterated di­methyl sulfoxide, h-DMSO [(CH_3_)_2_SO] and d-DMSO [(CD_3_)_2_SO]. The two different solvents, namely H_2_O and DMSO, were used to understand how hydrogen affects the shape and size of the resulting aggregates. We also report on the effect these solvents have on the aggregation behaviour, which highlights the importance of the colloidal nature of ND suspensions. The presence of *sp* ^2^ carbon and functional groups directly affects the stability of NDs in various media. Baidakova *et al.* (2013 ▸) reported that it is possible to produce stable suspensions of DNDs with agglomerates about 300 Å in size by appropriate methods of fractionation of water DND suspensions, and Avdeev *et al.* (2009 ▸) noted that liquid dispersions were stable for at least one year, which could be due to the presence of carb­oxy­lic acid groups. Colloidal stability is also a critical factor for the use of NDs as potential enterosorbents in drug delivery.

The availability of stable suspensions over a wide range of DND concentrations (up to the level of 10 wt%) makes it possible to apply the method of small-angle scattering of thermal neutrons, which is very efficient in structural research with its powerful option of contrast variation, as described above. This employs the changes in the scattered intensity arising from the different content of the deuterated component in the liquid carrier. In suspensions, the contrast variation is generated by dissolving the initial concentrated solutions in mixtures of light and heavy solvents (Avdeev *et al.*, 2009 ▸).

The contrast variation procedure was carried out by mixing the initial samples with appropriate mixtures of nondeuterated and deuterated solvents, making it possible to extract information on the inner structure of the aggregates. The SANS data from the liquid dispersion can also be compared with those obtained from the initial dry ND powder samples. Scattering from suspensions enables variation of the scattering length density (SLD) background as a contrast with the powder. Understanding the structure will allow better preparation of suspensions/samples for applications.

## 2. Experimental methods

The DND material was supplied as part of an IAEA collaborative research project. The LND material was obtained from Ray Techniques Ltd (Jerusalem, Israel) (Zousman & Levinson, 2014 ▸, 2012 ▸). Four dry powder sample types were studied, namely unheated and heated DNDs, and unheated and heated LNDs. The heating was carried out at 423 K for 24 h to remove any adsorbed water.

To perform the contrast variation procedure, suspensions were prepared with different mixtures of H_2_O/D_2_O and d-DMSO/h-DMSO. Suspensions were prepared at 0.5% by weight. All suspensions were ultrasonicated for 30 min (20 kHz) in an ultrasonic bath (using a probe, Sonics & Materials Inc., USA) at the time of preparation and again shortly before loading onto the instrument. Scattering length densities (ρ) have been calculated from chemical structure and physical density and are given in Table 1 ▸.

The neutron scattering experiments were conducted on the instruments located at Australia’s OPAL reactor (http://www.ansto.gov.au/ResearchHub/OurInfrastructure/ACNS/Facilities/Instruments/index.htm). The SANS measurements were performed on the QUOKKA instrument (Wood *et al.*, 2018 ▸) using three detector configurations to cover the *Q* range 0.0007–0.74 Å^−1^, using wavelengths of 5.0 and 8.1 Å. Low *Q* up to 0.0007 Å^−1^ was achieved by using lens optics with a wavelength of 8.1 Å. The configurations were *L*_1_ = *L*_2_ = 20 m (lens optics), *L*_1_ = *L*_2_ = 12 m and *L*_1_ = 12 m, *L*_2_ = 1.3 m (300 mm offset), where *L*_1_ and *L*_2_ are the source-to-sample and sample-to-detector distances, respectively. SANS data were reduced employing the NIST Center for Neutron Research SANS data reduction macros modified for the QUOKKA instrument, implemented in the *IGOR* software package (Kline, 2006 ▸), and transformed to an absolute scale following the use of an attenuated direct-beam transmission measurement (Nesvizhevsky, Dubois *et al.*, 2018 ▸). The USANS measurements were performed over a *Q* range of 1.8 × 10^−5^ to 0.052 Å^−1^ at a wavelength of 2.37 Å using the KOOKABURRA instrument (Rehm *et al.*, 2013 ▸). Solvent background was subtracted from the data. These data were desmeared using the NIST *IGOR* macros and then merged with the QUOKKA SANS data to give the full *Q* range. This broad *Q* range provides information on the structure of NDs on multiple length scales. The NDs tend to aggregate and form large structures, and neutrons provide additional information to other scattering techniques to understand the structure at different length scales. All measurements were made at room temperature.

## 3. Results and discussion

**Figure 2:**
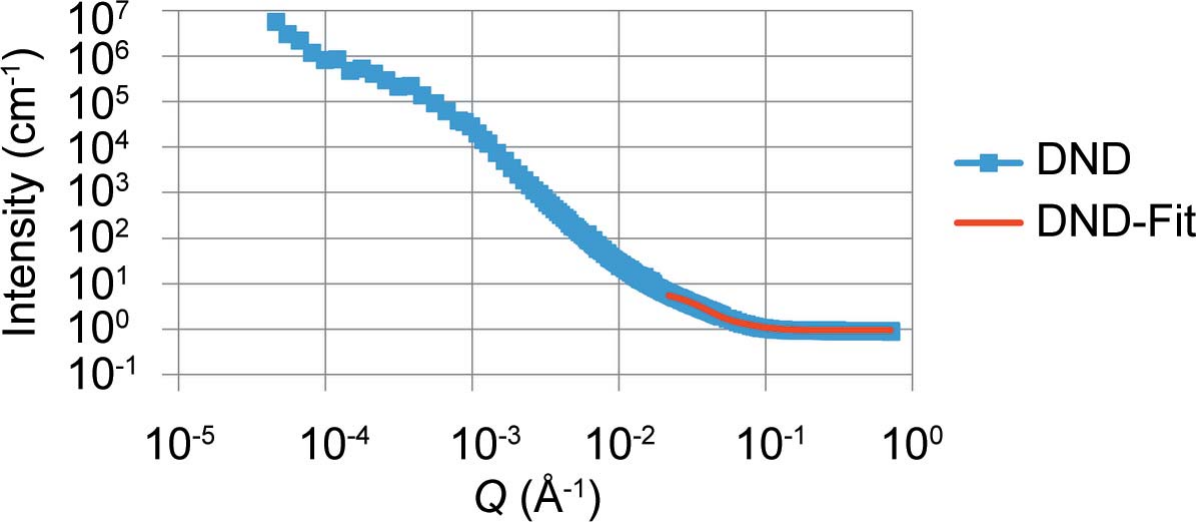
SANS plots of DND samples in H_2_O, with data in blue and the E-model fit in red. (Only data for *Q* > 0.022 Å^−1^ were used in the fitting.)

**Figure 3:**
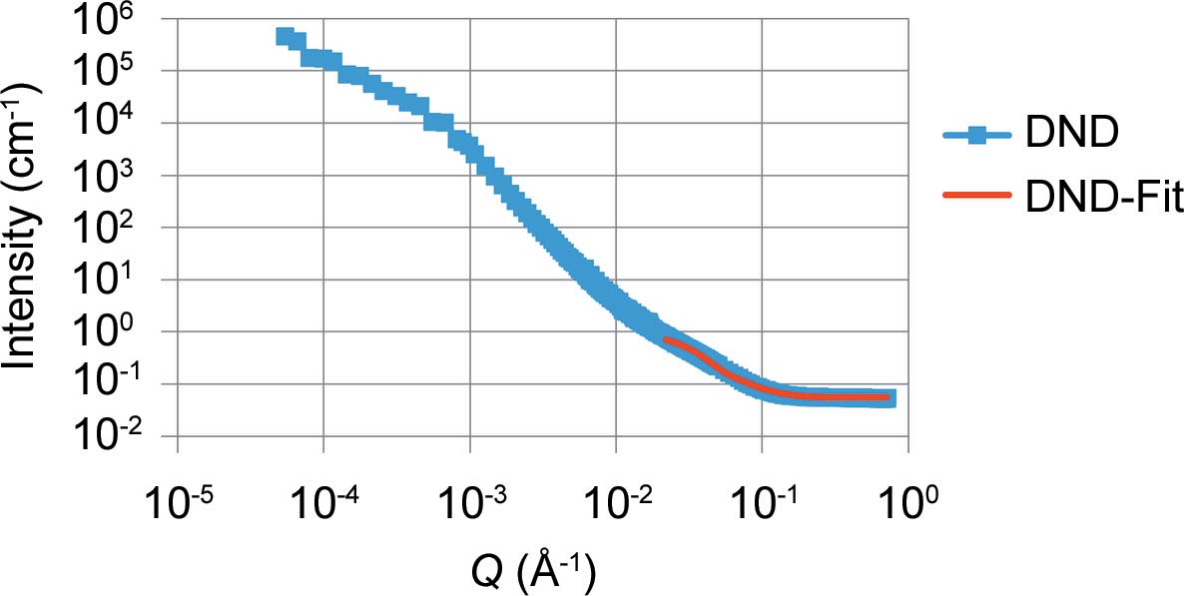
SANS plots of DND samples in D_2_O, with data in blue and the E-model fit in red. (Only data for *Q* > 0.022 Å^−1^ were used in the fitting.)

Data analysis was undertaken using the *SasView* program, Version 4.0.1 (http://www.sasview.org/). The merged data showed two shoulders and a power-law slope region for the initial dry ND powder samples. We have reported this approach previously (Ersez *et al.*, 2018 ▸). For easier interpretation the data were split into three regimes of *Q* ranges, namely low-*Q*, mid-*Q* and high-*Q* regions. For example, the low-*Q* to mid-*Q* regions in Figs. 2, 3 and 6–9 below (first shoulder region) provide information on the micrometre scale and the mid-*Q* to high-*Q* regions (*i.e.* the second shoulder region, Figs. 6–9) provide information on the nanoscale (Ersez *et al.*, 2018 ▸).

The Guinier–Porod model (GP model) developed by Hammouda (2010 ▸) is an empirical model applicable to objects of arbitrary shape and provides an estimate of the radius of gyration *R*_g_ and the dimensional variable *s*. Values of *s* determined in our previous work from combined USANS and SANS data (Ersez *et al.*, 2018 ▸) indicated ellipsoid-like objects for the dry powder samples and 3D rod-like objects for the other samples in the mid-*Q* to high-*Q* region. The scattering particle can be assumed to have an intermediate ellipsoidal structure between a sphere and a rod, and this was supported using the ellipsoid model (E model) to fit the mid-*Q* to high-*Q *region (2.2 × 10^−2^ ≤ *Q* ≤ 7.1 × 10^−1^ Å^−1^) (Figs. 2 ▸ and 3 ▸). In the E model the scattering intensity function for oriented ellipsoids is defined as follows (Feigin & Svergun, 1987 ▸):

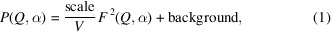

where

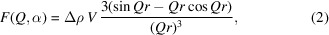

and

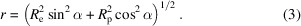

Here, α is the angle between the axis of the ellipsoid and the scattering vector **Q**, *V* = 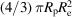 is the volume of the ellipsoid, where *R*_p_ is the polar radius along the rotational axis of the ellipsoid and *R*_e_ is the equatorial radius perpendicular to the rotational axis of the ellipsoid, and Δρ is the difference in the SLDs of the scatterer and the solvent.

**Figure 4:**
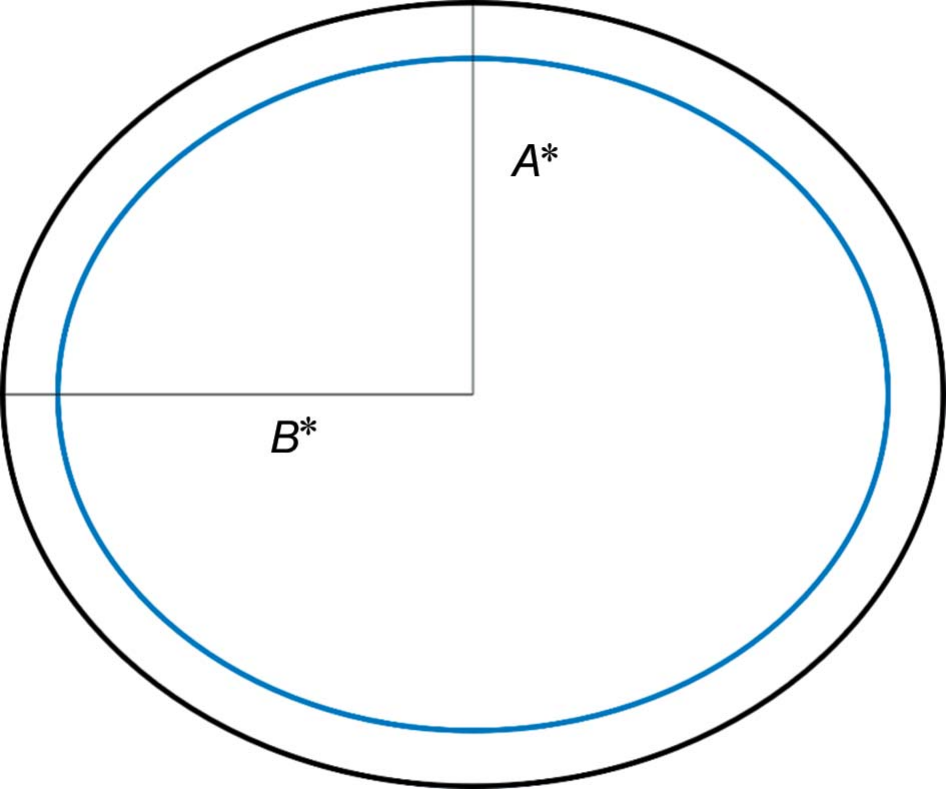
A representation of the ND core + shell structure, showing the polar radius A* (semi-minor axis) and the equatorial radius B* (semi-major axis).

If the radius *R*_e_ > *R*_p_, the object has an oblate ellipsoid form (disc like). If *R*_e_ < *R*_p_, the object is said to be a prolate ellipsoid (rod like). If *R*_e_ = *R*_p_ then the object is a sphere. In Fig. 4 ▸ the polar radius (semi-minor axis) is given by A* (core + shell) and the equatorial radius (semi-major axis) is given by B* (core + shell).

The E-model fitting parameters are given in Tables 2 ▸ and 3 ▸ for the DND and LND samples, respectively. The SLDs of the ND core and of the suspensions of NDs at various concentrations in H_2_O, D_2_O, h-DMSO and d-DMSO were fixed. The *R*_p_ and *R*_e_ values were found to be in the ranges 12–18 Å and 80–90 Å, respectively. The variation in *R*_p_ and *R*_e_ is most likely due to polydispersity. However, a spherical core was not considered here, and the omission of polydispersity as a parameter in the fitting has shown an effect on one of the axes.

**Figure 5:**
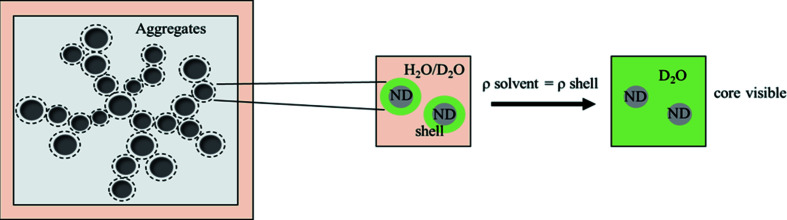
The application of contrast variation, showing the contrast match point for the shell.

For the samples in suspension the LND fitting parameters of *R*_p_ and *R*_e_ were slightly larger than those for the DNDs, but the decrease with decreasing contrast was of a similar amount. The fitted radii of the ND particles were found to be larger in H_2_O than in D_2_O, and similarly larger in h-DMSO than in d-DMSO. The decrease in radius was monotonic as a function of deuterium fraction in the case of *R*_p_ with H_2_O (both DNDs and LNDs). The observed difference in the radii of the ND particles with the contrast variation with hydrogen/deuterium substitutions in the dispersion media points to the existence of a shell. The difference between the fitted radius in deuterated and hydrogenous media may be regarded as an estimate of the shell thickness. The thickness estimated in this way is ∼5 Å (equatorially); previous values for the shell thickness in the literature have been given in the range 4–10 Å (Aleksenskii *et al.*, 2000 ▸; Palosz *et al.*, 2002 ▸; Avdeev *et al.*, 2013 ▸; Shah *et al.*, 2019 ▸). When the SLD of the solvent approaches that of the shell, the influence of the shell on the scattering decreases. The particle surface is composed of material which has an SLD value similar to that of D_2_O, and effectively one starts to see mainly the core particles which have a smaller radius (Aleksenskiy *et al.*, 2011 ▸; Avdeev *et al.*, 2009 ▸) (see Fig. 5 ▸). The SLDs of a number of materials, as calculated from the densities, are given in Table 1 ▸. These SLDs indicate that carbon black and graphite have similar values to D_2_O. So, moving in the direction away from the core, the shell becomes less dense, becoming similar in SLD to D_2_O.

Similar trends were shown in the *R*_p_ and *R*_e_ parameters from our earlier separate SANS measurements of DND and LND (Tables 4 ▸ and 5 ▸) in solvents of H_2_O, D_2_O(0.25)–H_2_O(0.75), D_2_O(0.5)–H_2_O(0.5), D_2_O(0.75)–H_2_O(0.25) and D_2_O. The changes in the scattering curves allowed analysis with a different content of the deuterated component in the liquid carrier. In these suspensions the LND fitting parameters of *R*_p_ and *R*_e_ were slightly larger than those of the DNDs, but the decrease with decreasing contrast was of a similar amount.

Therefore, using the E model the difference between the fitted radius in deuterated and hydrogenous media indicates that the non-diamond outer shell consists of carbonaceous and graphitic materials, which may be up to ∼5 Å thick (equatorially). The difference in SLD between the shell and the diamond core would be partly due to the lower physical density of the shell material relative to the core and partly due to the presence of a small fraction of hydrogen (whose SLD is negative) in the shell. Previously, Avdeev *et al.* (2009 ▸) estimated the effective thickness of the non-diamond shell in DNDs to be about 5 Å, with the whole particle of ∼70 Å composed of a crystalline diamond core and a graphene-like shell. Tomchuk *et al.* (2011 ▸) also suggested that in their samples there is a graphene shell around the diamond core. Similarly, they observed a decrease in the radii of gyration for subparticles with decreasing contrast from 4.6 (8) to 2.8 (5) nm and the scattering decayed monotonically with increasing deuterium content.

For typical small-angle scattering situations there may be a number of closely similar ‘best fits’, so in order to reduce the number of floating parameters the E model was also used by fixing the *R*_p_ parameters at average values between the DND and LND samples [*e.g.* average (DND and LND) *R*_p_ = 12.8 Å from Tables 2 ▸ and 3 ▸]. The *R*_e_ parameters were allowed to vary. The fit was performed individually for each concentration. The fitting parameters for these radii are given in Tables 6 ▸ and 7 ▸.

We now consider the entire *Q* range including both USANS and SANS measurements. USANS measurements were carried out on dry powders and on suspensions in H_2_O, D_2_O and h-DMSO.

**Figure 6:**
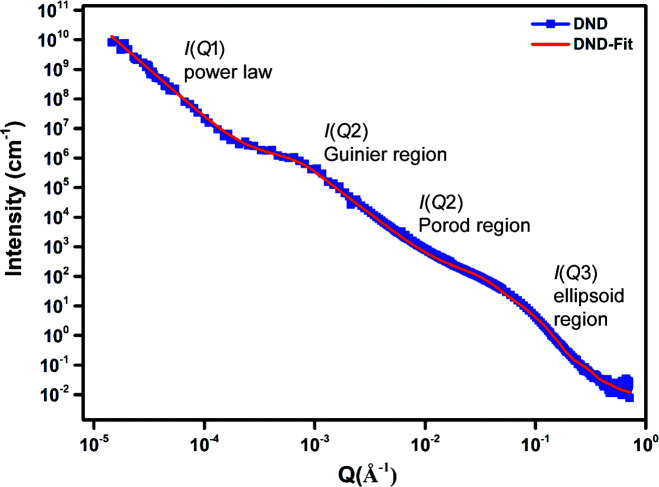
USANS/SANS plots of unheated dry powder DND samples.

**Figure 7:**
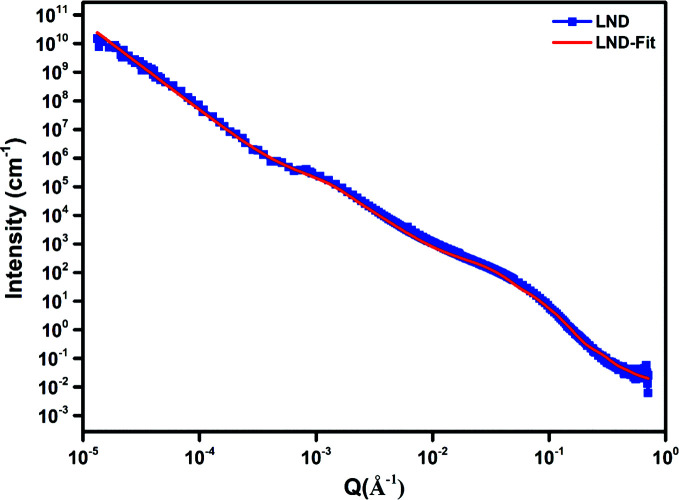
USANS/SANS plots of unheated dry powder LND samples.

The low-*Q* region (2.0 × 10^−5^ ≤ *Q* ≤ 1.4 × 10^−4^ Å^−1^), mid-*Q* region (1.4 × 10^−4^ ≤ *Q* ≤ 2.2 × 10^−2^ Å^−1^) and high-*Q* region (2.2 × 10^−2^ ≤ *Q* ≤ 7.1 × 10^−2^ Å^−1^) are designated *I*(*Q*1), *I*(*Q*2) and *I*(*Q*3), respectively, as shown in Fig. 6 ▸. In region *I*(*Q*1) the power-law function was used, in region *I*(*Q*2) the GP model was used and in region *I*(*Q*3) the E model was used. The rationale of using these models relies mainly on the fact that NDs have a well defined core–shell structure which we have modelled using ellipsoids. This allowed us to limit the number of parameters so we can determine the shell thickness indirectly with high confidence. For the mid-*Q* region, aggregations are formed randomly due to ND interactions. We wanted to capture this by applying a simple empirical model. The *R*_g_ and *s* values are not interpreted literally but instead used as parameters to compare the various samples. This approach has been used previously (Shah *et al.*, 2019 ▸). At low *Q*, the scattering continues beyond the 20 µm length scale and resembles mass fractal structures. This is captured by applying a power-law model and comparing the slope values for different mixtures. The parameters obtained from the fits for these three regions provided useful information for further modelling of the USANS and SANS data. Next, the whole *Q* range for the combined USANS and SANS data was fitted using a custom plugin model created in *SasView* (Hammouda, 2010 ▸; Feigin & Svergun, 1987 ▸; Aleksenskii *et al.*, 2000 ▸; Palosz *et al.*, 2002 ▸; Avdeev *et al.*, 2013 ▸; Shah *et al.*, 2019 ▸; Kim & Glinka, 2006 ▸; Hjelm *et al.*, 1994 ▸), comprising a power-law function + Guinier–Porod + ellipsoid (thus termed a PGE model). This custom model is defined as

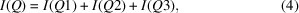

where *I*(*Q*1) is the power-law function, *I*(*Q*2) is the GP model and *I*(*Q*3) the E model.

**Figure 8:**
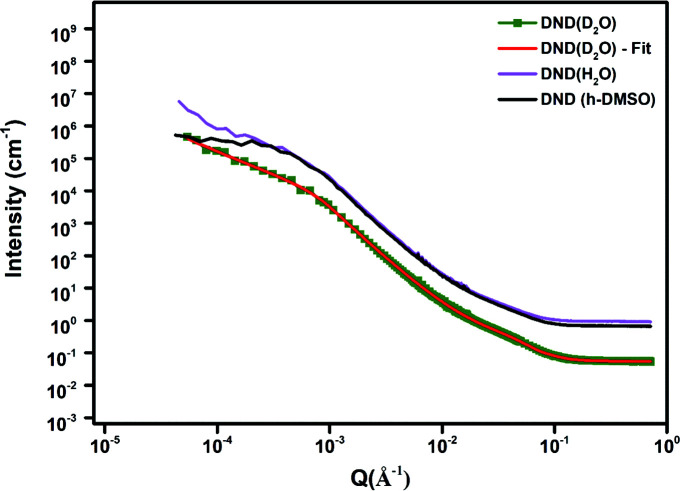
USANS/SANS plots of DND samples in H_2_O, D_2_O and h-DMSO.

**Figure 9:**
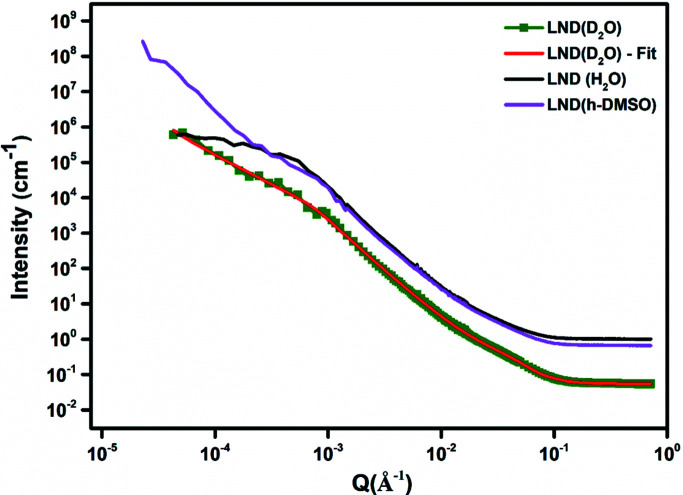
USANS/SANS plots of LND samples in H_2_O, D_2_O and h-DMSO.

For the PGE model the parameters *R*_g_ (for the GP model at low *Q*), scale factor (for the E model at high *Q*), *R*_p_ and *R*_e_ were allowed to vary. The models reproduce both the overall intensity and the undulating features seen in the high-*Q* range. Figs. 6 ▸ and 7 ▸ show the unheated dry DND and LND fitted data and Figs. 8 ▸ and 9 ▸ the data for NDs in suspension. A monotonic decrease in the absolute scattered intensity is observed, with insignificant changes in the character of the curves. Good fits were obtained with this model, and the resulting parameters are shown in Tables 6 ▸ and 7 ▸. The GP model was used to obtain initial estimates for the *R*_g_ parameters. The USANS/SANS data have revealed dimensions (*R*_g_ values) up to 3000 Å (low *Q*). As each sample is expected to have a distribution of particle sizes and shapes, the *R*_g_ value should not be seen as the actual size of each aggregate, but rather as a parameter which allows us to make a comparison between different samples.

The observed aggregates in the liquid suspensions differ from the initial dry powder samples (the sizes of the aggregates are given in Tables 6 ▸ and 7 ▸). It may be seen that the value of *R*_g_ in a suspension is in some cases lower and in other cases higher than in a dry powder. This is likely to be due to a combination of factors. Ultrasonication when the suspensions were initially prepared may have broken up some aggregates and thus reduced the average size, but as the suspensions were left to stand for some time, aggregates may have grown by the merger of smaller aggregates or as any free particles became attached to aggregates. At low *Q* the power-law slope of the different samples is in the range 0.7–5.2. The rise at low *Q* can be said to be due to the fractal nature of the aggregates. The fractal dimensionality, *D*, is defined as *n* = 6 − *D* for 3D objects, where *n* is the power-law index (Kim & Glinka, 2006 ▸). At low *Q* the power-law slopes for the dry powder ND samples suggest fractal surfaces with dimensionality of *D* = 3.0–3.3 and in the suspensions *D* = 0.8–5.3. For the DND and LND samples in H_2_O the fitting parameters are very similar, except for the value of the power-law slope (LNDs much less than DNDs). The outer radii of the ellipsoids are the sum of the corresponding core radius and shell thickness. Using the PGE model also gives smaller radii for NDs in D_2_O suspensions than in H_2_O and h-DMSO. Treating the difference between *R*_e_ in H_2_O and D_2_O as an estimate of the thickness of the carbonaceous and graphitic layer, this model estimates a thickness of ∼6 Å, slightly larger than that obtained above with the E model and within the shell thickness range of 4–10 Å given in the literature (Aleksenskii *et al.*, 2000 ▸; Palosz *et al.*, 2002 ▸; Avdeev *et al.*, 2013 ▸). In Figs. 8 ▸ and 9 ▸ only data using the D_2_O solvent are shown with the model fit (red). The magenta curve shows the data using H_2_O and the black curve shows the data for the h-DMSO solvent. In both cases, no model fit is shown overlaid on the data plot. It seems the data using the h-DMSO solvent show a steeper slope at low *Q* compared with those of the other two solvents.

## 4. Conclusions

At the nanoscale level, the ND particles were assumed to have an intermediate ellipsoidal structure between spheres and rods. The full-*Q*-range fit provided a consistency check of the *Q* sectioning using the Hammouda model, and so the initial choice (Figs. 2 ▸ and 3 ▸) of the high-*Q* end was found to be reasonable. The DNDs and LNDs in suspension demonstrated a consistent oblate ellipsoidal structure, with particles having *R*_p_ and *R*_e_ values in the ranges 12–18 Å and 80–90 Å, respectively. Using a defined shape model and the (U)SANS contrast variation technique, it was possible to characterize the non-diamond outer shell of the DND and LND particles as comprising a carbonaceous and graphitic type material, by analysis of the changes in SLD from the variation in the SLDs of the solvents (H_2_O, D_2_O, h-DMSO or d-DMSO). The shell was estimated to have a thickness of ∼6 Å (in the equatorial region).

The USANS/SANS data have also revealed the dimensions of aggregates up to 3000 Å (low *Q*). The observed aggregates in the liquid suspensions differ from the initial dry powder samples. The structures of the aggregates observed using SANS data are similar for the liquid carriers H_2_O and h-DMSO, while H_2_O and D_2_O show differences, mainly at low *Q*. This information will be useful for possible modification and enhancement of the properties and characteristics of ND particles through surface modification and will enable them to play a major role in the development of high-albedo materials for VCN reflection, by controlling the leakage of cold and very cold neutrons in a moderator.

The proposed application of nanodiamond materials as a reflector of very cold neutrons requires the ND particles to have dimensions similar to the neutron wavelength. While the individual ND particles have in fact been shown to have a size range commensurate with VCN wavelengths, the aggregates are considerably larger than this. The high-*Q* part of the SANS data shows that the individual particles within the aggregates are visible to neutrons, so it is expected that aggregation will not prevent the use of NDs as a VCN reflector. However, aggregation should be taken into account in the modelling of VCN reflection, to assess whether the efficiency of reflection is affected by this. The aggregate size of dry powder ND mater­ials has been found to be significantly smaller for LNDs than for DNDs, so if aggregation reduces reflection efficiency it would be preferable to use LNDs.

For pharmaceutical applications of NDs, aggregation is a significant disadvantage, since particles near the centre of an aggregate may not be readily accessible to reagents for the addition of pharmaceutically active functional groups. Such applications would typically require liquid-phase chemical reactions in order to add these functional groups, and our results indicate that in some cases the aggregation may increase when a suspension of NDs in a liquid is prepared. Consequently, it may not be sufficient to minimize aggregation in the initial dry powder form of NDs – there may be a need to deal with aggregation which develops in the liquid suspension. Several procedures for deagglomeration of ND aggregates have been proposed, such as milling with beads (Krueger *et al.*, 2007 ▸).

Aggregation is probably caused, at least in part, by van der Waals interactions between neighbouring ND particles (Xu & Zhao, 2012 ▸). However, hydrogen present in the shells of ND particles may also play a part in aggregation. NMR studies (Fang *et al.*, 2009 ▸) have shown that some of this hydrogen exists in the form of hydroxyl groups. A hydroxyl group may form a hydrogen bond with an oxygen atom in an adjacent ND particle, thus contributing to aggregation. The thermal treatment (423 K) and outgassing of ND samples was found to eliminate hydrogen in the form of water adsorbed on the nanoparticle surface, but hydrogen bonded to carbon was difficult to remove (Ersez *et al.*, 2018 ▸; Krylov *et al.*, 2011 ▸). However, this preliminary thermal treatment and outgassing of samples does not significantly improve the reflecting properties of ND powders. It would be interesting to compare hydrogenous and deuterated NDs in order to check whether the deuterium is bonded in the same way as the hydrogen which it has replaced. The much lower neutron cross section of D compared with H can prove to be very useful in that the position of H in the crystal structure and its thermal motions can be determined with greater precision. Recently, Bosak *et al.* (2020 ▸) observed that the replacement of H by F in their ND powder samples increased the neutron reflectivity due to the large reduction in neutron losses.

Understanding aggregation is required before using ND powders as reflectors for VCNs. This will also play an important role in the fields of other carbon-containing interfaces, such as in medicine.

## Figures and Tables

**Table 1:** Neutron scattering length densities (SLDs) ρ of various materials, including solvents, used in this work

Material	ρ (× 10^−6^ Å^−2^)	Physical density (g cm^−3^)
Carbon black (Kim & Glinka, 2006 ▸)	6.2	1.91
Graphite	7.55	2.266
Crystalline diamond	11.73	3.520
C_2_H_5_OH	−0.35	0.789
C_2_D_5_OD	6.16	0.901
H_2_O†	−0.5595	0.998
D_2_O(0.33)–H_2_O(0.67)†	1.749	
D_2_O(0.67)–H_2_O(0.33)†	4.058	
D_2_O†	6.366	1.105
h-DMSO†	−0.0419	1.096
d-DMSO(0.33)–h-DMSO(0.67)†	1.731	
d-DMSO(0.67)–h-DMSO(0.33)†	3.505	
d-DMSO†	5.278	1.190

† Prepared in this work.

**Table 2:** SANS ellipsoid model parameters for the DND samples

Sample	*R*_p_ (Å) (DND)	Error	*R*_e_ (Å) (DND)	Error	Scale
H_2_O	15.2	0.1	83.5	0.1	0.00107
D_2_O(0.33)–H_2_O(0.67)	14.2	0.2	87.2	0.3	0.00086
D_2_O(0.67)–H_2_O(0.33)	13.9	0.1	83.3	0.2	0.00110
D_2_O	11.8	0.1	80.2	0.2	0.00113
h-DMSO	15.0	0.1	83.4	0.2	0.00106
d-DMSO(0.33)–h-DMSO(0.67)	13.0	0.02	85.0	0.2	0.00110
d-DMSO(0.67)–h-DMSO(0.33)	14.5	0.02	80.7	0.2	0.00103
d-DMSO	13.8	0.1	79.2	0.2	0.00094
Dry, unheated	17.3	0.03	80.1	0.09	0.03700
Dry, heated	18.0	0.03	80.2	0.09	0.03700

**Table 3:** SANS ellipsoid model parameters for the LND samples

Sample	*R*_p_ (Å) (LND)	Error	*R*_e_ (Å) (LND)	Error	Scale
H_2_O	15.5	0.1	90.7	0.2	0.00102
D_2_O(0.33)–H_2_O(0.67)	15.3	0.2	83.0	0.2	0.00086
D_2_O(0.67)–H_2_O(0.33)	14.7	0.1	86.7	0.2	0.00110
D_2_O	13.8	0.1	85.6	0.2	0.00102
h-DMSO	16.5	0.1	87.3	0.2	0.00100
d-DMSO(0.33)–h-DMSO(0.67)	11.5	0.02	90.3	0.2	0.00144
d-DMSO(0.67)–h-DMSO(0.33)	14.7	0.02	85.9	0.2	0.00118
d-DMSO	15.7	0.1	83.4	0.2	0.00090
Dry, unheated	17.3	0.04	80.2	0.08	0.05600
Dry, heated	17.7	0.03	80.3	0.07	0.07000

**Table 4:** SANS ellipsoid model parameters for the DND samples in solvents

Sample	*R*_p_ (Å) (DND)	Error	*R*_e_ (Å) (DND)	Error	Scale
H_2_O	15.8	0.2	84.9	0.3	0.00119
D_2_O(0.25)–H_2_O(0.75)	15.5	0.3	85.1	0.4	0.00111
D_2_O(0.50)–H_2_O(0.50)	15.2	0.3	83.4	0.4	0.00111
D_2_O(0.75)–H_2_O(0.25)	14.1	0.3	83.4	0.4	0.00118
D_2_O	14.5	0.3	81.2	0.4	0.00096

**Table 5:** SANS ellipsoid model parameters for the LND samples in solvents

Sample	*R*_p_ (Å) (LND)	Error	*R*_e_ (Å) (LND)	Error	Scale
H_2_O	17.3	0.2	89.9	0.3	0.00107
D_2_O(0.25)–H_2_O(0.75)	16.8	0.2	90.2	0.4	0.00109
D_2_O(0.50)–H_2_O(0.50)	16.4	0.3	89.4	0.4	0.00110
D_2_O(0.75)–H_2_O(0.25)	15.5	0.3	89.7	0.4	0.00117
D_2_O	16.1	0.2	86.6	0.4	0.00102

**Table 6:** USANS/SANS model parameters for the DND samples PGE denotes the combined power law, GP and E models. E denotes the E model (the polar radius is fixed in this case).

Parameter	DND (unheated, dry)	Error	DND (H_2_O)	Error	DND (D_2_O)	Error	DND (h-DMSO)	Error
*R*_g_ (Å)	1982.8	65.7	2064.8	87.3	1145.2	88.0	1790.6	43.4
*s*	0.15	0.06	0.91	0.05	1.27	0.09	0.67	0.02
Porod	2.84	0.003	2.89	0.0003	3.33	0.003	2.90	0.005
Power-law index	3.25	0.01	5.17	0.04	2.30	0.02	0.66	0.54
Polar radius (PGE) (Å)	17.6	0.04	15.8	0.2	15.2	0.4	16.0	0.8
Equatorial radius (PGE) (Å)	80.5	0.1	67.5	0.2	61.5	0.4	68.6	0.2
Polar radius (E) (Å)	17.5	Fixed	15.5	Fixed	12.8	Fixed	15.5	Fixed
Equatorial radius (E) (Å)	79.9	0.09	83.3	0.1	79.7	0.2	83.1	0.2

**Table 7:** USANS/SANS model parameters for the LND samples PGE denotes the combined power law, GP and E models. E denotes the E model (the polar radius is fixed in this case).

Parameters	LND (unheated, dry)	Error	LND (H_2_O)	Error	LND (D_2_O)	Error	LND (h-DMSO)	Error
*R*_g_ (Å)	1075.7	71.9	2862.5	83.7	1277.9	110.2	1727.1	291.5
*s*	0.5	0.1	0.33	0.05	1.21	0.09	1.01	0.22
Porod	2.6	0.003	2.88	0.01	3.10	0.03	2.65	0.01
Power-law index	3.06	0.01	1.96	0.03	2.26	0.02	3.23	0.06
Polar radius (PGE) (Å)	17.8	0.08	24.5	0.6	20.7	0.5	20.2	0.2
Equatorial radius (PGE) (Å)	83.6	0.1	67.56	0.4	60.9	0.4	63.8	0.3
Polar radius (E) (Å)	17.5	Fixed	15.5	Fixed	12.8	Fixed	15.5	Fixed
Equatorial radius (E) (Å)	80.1	0.07	90.7	0.2	86.2	0.2	87.9	0.1
